# Efficacy of transcranial direct current stimulation on motor and cognitive functions in patients with multiple sclerosis: a systematic review and meta-analysis

**DOI:** 10.1186/s12883-026-04961-y

**Published:** 2026-05-15

**Authors:** Ghazi Uddin Ahmed, Hussain Abbas, FNU Komal, FNU Muskan, Anusha Tharwani, Rithik Kumar Khiani, Saini Ramani, Ahmed Asad Raza, Jawad Ghias Shaikh, Owais Sanaullah, Abedin Samadi

**Affiliations:** 1 Department of Medicine, Jinnah Sindh Medical University, Karachi, Pakistan; 2 Department of Medicine, Kabul University of Medical Sciences Abu Ali Sina, Kabul, Afghanistan

**Keywords:** Transcranial direct current stimulation, Multiple sclerosis, Motor function, Cognitive function, Non-invasive brain stimulation

## Abstract

**Background:**

Multiple Sclerosis (MS) is a chronic neurological disorder affecting 2.8 million individuals worldwide, characterized by motor dysfunction and cognitive impairment that remain poorly addressed by pharmacological interventions alone. Transcranial Direct Current Stimulation (tDCS) has emerged as a promising non-invasive neuromodulation technique for symptom management in MS patients.

**Objectives:**

This systematic review and meta-analysis aimed to evaluate the efficacy of tDCS in improving motor and cognitive functions in patients with MS, and to assess its safety profile.

**Methods:**

A comprehensive literature search was conducted across PubMed, Scopus, EMBASE, Wiley Online Library, and Google Scholar databases. Studies were selected based on PICOS criteria, including randomized controlled trials and quasi-experimental studies involving adults (≥ 18 years) with all types of MS receiving tDCS interventions. Methodological quality was assessed using RoB 2.0 and ROBINS-I. Meta-analyses were performed using RevMan 5.4.1 with random-effects models for outcomes with substantial heterogeneity and fixed-effects models where I^2^ = 0%. Additionally, the protocol was prospectively registered with the Open Science Framework.

**Results:**

Twenty-two studies published between 2015 and 2025 were included in this review. Meta-analysis revealed that tDCS significantly improved information processing speed (SDMT: MD = 7.71, 95% CI: 1.60—13.82, *p* = 0.01) and functional mobility (TUG: MD = -1.03 s, 95% CI: -2.08—0.02, *p* = 0.05). While individual studies showed improvements in gait speed and balance, pooled analyses for these outcomes did not reach statistical significance (gait speed: MD = 0.16 m/s, 95% CI: -0.07—0.38, *p* = 0.18; Berg Balance Scale: MD = 1.18, 95% CI: -2.03—4.39, *p* = 0.47). Qualitative analysis revealed consistent improvements in manual dexterity, working memory, executive function, and complex attention. Additionally, no serious adverse events were reported across studies; mild and transient side effects (e.g., tingling, itching) were noted, and completion rates were high (98% in studies that reported them).

**Conclusion:**

This systematic review provides preliminary evidence supporting tDCS as a potentially beneficial adjunctive intervention for MS patients, particularly for cognitive processing speed enhancement based on two small studies. While individual studies reported motor improvements, pooled meta-analyses for gait speed and balance failed to demonstrate statistical significance, indicating insufficient evidence for definitive motor benefits despite positive signals in individual trials. The substantial limitation of small study numbers per meta-analysis (2—6 studies), combined with limited MS subtype-specific data in most studies, significantly constrains confidence in these findings and limits assessment of external validity across different MS populations. Larger, standardized, multi-center randomized controlled trials with adequate subgroup representation and extended follow-up periods are essential to establish clinical significance and treatment durability.

## Introduction

Multiple Sclerosis (MS) is a chronic, immune-mediated neurological disorder of the central nervous system characterized by inflammation, demyelination, and progressive neurodegeneration [27, 36]. Multiple Sclerosis affects an estimated 2.8 million individuals worldwide [2], and the disease course varies across subtypes, including relapsing–remitting MS (RRMS), primary progressive MS (PPMS), and secondary progressive MS (SPMS), with most patients initially presenting with RRMS [52]. While disease-modifying therapies have transformed the long-term management of MS by targeting inflammation and relapse rates, residual symptoms such as motor impairment and cognitive decline remain prevalent and poorly addressed by pharmacologic means alone [49].

Motor dysfunction is a prominent and often debilitating feature of MS. It encompasses a range of symptoms, including impaired mobility, gait abnormalities, spasticity, limb weakness, and reduced manual dexterity [17]. These deficits are frequently progressive and contribute to disability, reduced independence, and diminished quality of life. Impaired gait and poor hand coordination directly interfere with daily activities [7]. Alongside motor symptoms, cognitive impairment is observed in 40% to 65% of individuals with MS, even in early stages of the disease [3]. Key cognitive domains affected include attention, executive functioning, processing speed, and working memory [38].

The complexity and multifactorial nature of MS pathology have motivated the exploration of complementary treatment approaches aimed at symptom management and functional restoration. One of them, Transcranial Direct Current Stimulation (tDCS), has already shown potential as a non-invasive neuromodulation method. This technique delivers low-amplitude electrical current (typically 1–2 mA) to targeted cortical regions via scalp electrodes, thereby modulating neuronal excitability and inducing neuroplasticity [11]. Specifically, anodal stimulation increases neuronal excitability by reducing the resting membrane potential threshold, while cathodal stimulation exerts inhibitory effects,these polarity-dependent changes modulate functionally significant brain networks through mechanisms analogous to long-term potentiation and depression [24]. Concerning MS, tDCS has also proven promising in treating motor and cognitive symptoms. For motor rehabilitation, tDCS is typically applied over the primary motor cortex (M1), which is the preferred montage for targeting corticospinal motor pathways and enhancing gait, balance, and upper limb coordination, particularly in patients with RRMS and progressive subtypes exhibiting residual motor cortex plasticity [26]. In contrast, cerebellar montages are increasingly employed when ataxia, postural instability, or cerebellar involvement is prominent, as in some PPMS patients, given the cerebellum’s role in motor coordination and balance [1]. Concurrently, cognitive treatments usually include dorsolateral prefrontal cortex (DLPFC) stimulation, which is linked to executive functioning and working memory, and is applied irrespective of MS subtype when cognitive impairment is the primary target [4]. Several studies in a limited body of literature have found functional improvements following tDCS, such as increased walking speed, increased manual dexterity, and improved performance on cognitive tasks [14, 33, 53]. Despite these encouraging results, the clinical applicability of tDCS in MS has been controversial because of the heterogeneous methods in studies, limited sample size of studies, inconsistent stimulation parameters, and diverse outcome measurements. Moreover, variability in MS subtype, disease duration, and disability level complicates the interpretation of results. A critical methodological limitation of the existing literature is the frequent merging of MS subtypes (RRMS, PPMS, SPMS) within single trials, which prevents definitive subtype-specific conclusions. Therefore, this systematic review and meta-analysis attempt to address this gap by systematically evaluating the effect of transcranial direct current stimulation on motor and cognitive functions in multiple sclerosis patients,where data permitted, subtype-specific patterns are described, and the inability to perform formal subgroup meta-regressions by MS subtype is explicitly acknowledged as a major limitation.

### Objectives

The specific objectives of this review are:

1. To investigate the effectiveness of tDCS in enhancing motor functions in people with MS, such as improving gait speed and balance.
2. To evaluate the effect of tDCS on the cognitive functioning of MS patients, with a look at working memory, attention, executive functioning, and processing speed domains.
3. To identify the tolerability, adverse effect profile, and safety of tDCS in the population.

## Methodology

This study was reported according to the Preferred Reporting Items for Systematic Reviews and Meta-Analysis (PRISMA) [39], to ensure transparency and methodological rigour throughout the review process. Moreover, this systematic review was conducted according to a protocol prospectively registered with the Open Science Framework (OSF) before study commencement (Registration 10.17605/OSF.IO/89YCK). Protocol registration was undertaken to ensure methodological transparency and minimize potential selection bias in study identification and data extraction processes.

### Identification and selection of studies

An extensive search of the literature was conducted to find peer-reviewed original research studies investigating the effects of transcranial direct current stimulation (tDCS) on performance related to motor and cognitive activities in multiple sclerosis (MS) patients. The initial literature search was conducted on 29/06/2025 across electronic databases including PubMed, Scopus, EMBASE, Wiley Online Library, and Google Scholar. Google Scholar was included as a supplementary source to capture grey literature and ensure comprehensive coverage, following guidance by Haddaway et al. [20] and Godin et al. [18]. This supports its use for systematically identifying additional relevant literature in evidence reviews.

#### Search strategy

A systematic literature search was conducted across five electronic databases: PubMed, Scopus, Embase, Wiley Online Library, and Google Scholar. The search strategy was developed using a combination of Medical Subject Headings (MeSH) terms and keywords related to transcranial direct current stimulation and multiple Sclerosis, connected with Boolean operators (AND, OR), and a complete database-specific search string, and results are provided in Table 1. For Google Scholar, results were screened through the first 200 records per search string based on relevance ranking, in line with guidance from Haddaway et al. [20].

**Table 1:** Database-specific search results

Database	Search Strategy
PubMed	("transcranial direct current stimulation"[MeSH Terms] OR "transcranial direct current stimulation"[Title/Abstract] OR tDCS[Title/Abstract]) AND ("multiple sclerosis"[MeSH Terms] OR "multiple sclerosis"[Title/Abstract])
Scopus	TITLE-ABS-KEY("transcranial direct current stimulation") AND TITLE-ABS-KEY("multiple sclerosis")
EMBASE	"transcranial direct current stimulation" AND "multiple sclerosis" AND ("motor function" OR "gait" OR "balance") AND ("cognitive function" OR "working memory" OR "processing speed") AND "Clinical trials"
Wiley Online Library	"transcranial direct current stimulation" AND "multiple sclerosis" AND ("motor function" OR gait OR balance OR "manual dexterity") AND ("cognitive function" OR "working memory" OR "executive function" OR "processing speed") AND ("clinical trial" OR "randomized controlled trial")
Google Scholar	"transcranial direct current stimulation" AND "multiple sclerosis" AND ("motor function" OR "gait" OR "balance") AND ("cognitive function" OR "working memory" OR "processing speed") AND "Clinical trials"

#### Study selection

Two independent reviewers screened the titles and abstracts of retrieved records to identify potentially eligible studies. Full-text articles of potentially relevant studies were subsequently reviewed to determine final inclusion. Inter-reviewer agreement was assessed using Cohen's kappa statistic (κ = 0.82 for title/abstract screening and κ = 0.88 for full-text screening), indicating almost perfect agreement. Disagreements were resolved through discussion between reviewers, with a third reviewer consulted when consensus could not be reached. All references were managed using Zotero version 6.0.36, which performed automated screening for retracted studies by cross-referencing articles against known retraction databases and flagged potential duplicate records. The retraction screening identified no retracted articles among the included studies, and duplicate entries flagged by the software were manually reviewed and merged by one reviewer, with verification by a second reviewer to ensure accuracy.

### Eligibility criteria

#### Inclusion criteria

The inclusion criteria were established based on the PICOS (Population, Intervention, Comparison, Outcomes, Study Design) framework [35]:

- Population: Adults (≥ 18 years) diagnosed with Multiple Sclerosis (MS), including relapsing–remitting, primary progressive, and secondary progressive forms.
- Intervention: Transcranial Direct Current Stimulation (tDCS), applied alone or as an adjunct to other therapies.
- Comparison: Sham stimulation, no stimulation, or other standard interventions.
- Outcomes:
  - Primary outcomes: Changes in motor function (e.g., gait speed, balance, limb coordination) and cognitive function (e.g., memory, attention, executive functioning, processing speed).
  - Secondary outcomes: Safety and tolerability, reported adverse events, and dropout rates.
- Study Design: Randomized Controlled Trials (RCTs) and quasi-experimental clinical trials.

#### Exclusion criteria

The following were excluded from the review:

- Non-original research articles (e.g., letters, editorials, opinion pieces)
- Conference abstracts, protocols, and reviews (including systematic reviews and meta-analyses)
- Studies not published in English (this restriction may introduce language bias, which is acknowledged as a limitation of this review)
- Studies without available full-text (i.e., studies for which the full text was not retrievable by the review team regardless of open-access status; studies accessible only via institutional subscription were included if retrieved through authors’ institutional access)

### Methodological quality assessment

The risk of bias in the eligible studies was assessed using tools appropriate to the study design. For non-randomized clinical trials, the ROBINS-I tool, visualized using the robvis tool, was used to determine bias across seven domains: confounding, selection of participants, classification of interventions, deviations from intended interventions, missing data, measurement of outcomes, and selection of the reported result [34]. On the other hand, for the randomized controlled trial, the RoB 2.0 tool, also visualized using RobVis, was applied to evaluate bias in five domains: randomization process, deviations from intended interventions, missing outcome data, measurement of the outcome, and selection of the reported result [34]. All studies were included in the analysis regardless of risk of bias rating. Studies with high risk of bias were clearly identified in the results tables, and their limitations were discussed when interpreting findings.

### Data extraction

Data from included studies were extracted using a pre-designed Microsoft Excel 2019 spreadsheet. The first reviewer performed the extraction, and the second independently verified the entries for accuracy and completeness. Extracted data included:

- Study identification details (authors, year)
- Sample size, characteristics, and MS subtype
- Intervention Parameters (Type of tDCS, Electrode placement, Intensity, Session duration/number)
- Target site
- Outcome measures
- Key results
- Reported adverse events and dropout rates

### Data analysis

#### Qualitative synthesis

Two independent reviewers narratively synthesized data from studies not eligible for quantitative synthesis and systematically extracted and grouped them according to outcome domains (motor function, cognitive function, safety outcomes). Both reviewers conducted Initial coding independently, followed by collaborative theme development through iterative discussion. Inter-rater agreement for coding was assessed using percentage agreement, and a synthesis process was performed manually without qualitative analysis software. The coded themes were then checked for consistency [12].

#### Quantitative synthesis (Meta-Analysis)

A meta-analysis was conducted using Review Manager (RevMan, version 5.4.1) for studies with sufficient quantitative data. Continuous outcomes were analyzed using the inverse variance method, and results were expressed as mean differences (MDs) with corresponding 95% confidence intervals (CIs). Model selection was based on the degree of heterogeneity: random-effects models were applied for outcomes with substantial heterogeneity (I^2^ ≥ 50%), while fixed-effects models were used for outcomes with low or absent heterogeneity (I^2^ = 0%), consistent with established methodological guidance [21]. Specifically, random-effects models were used for TUG (I^2^ = 79%) and BBS (I^2^ = 35%), while fixed-effects models were used for gait speed (I^2^ = 0%) and SDMT (I^2^ = 0%). All meta-analyses compared endpoint values between active tDCS and sham groups,where studies reported change-from-baseline scores, these were used in place of endpoint scores. Mixing of endpoint and change scores was avoided where possible; any exceptions are noted in the individual forest plot descriptions.

#### Publication bias assessment

Publication bias was assessed through funnel plot analysis by examining the distribution pattern of studies around the pooled effect estimate. According to established methodological guidelines, visual assessment of funnel plots provides limited reliability with fewer than 10 studies, as statistical power for detecting asymmetry diminishes substantially with smaller study numbers [21].

## Results

### Study selection

**Fig. 1:**
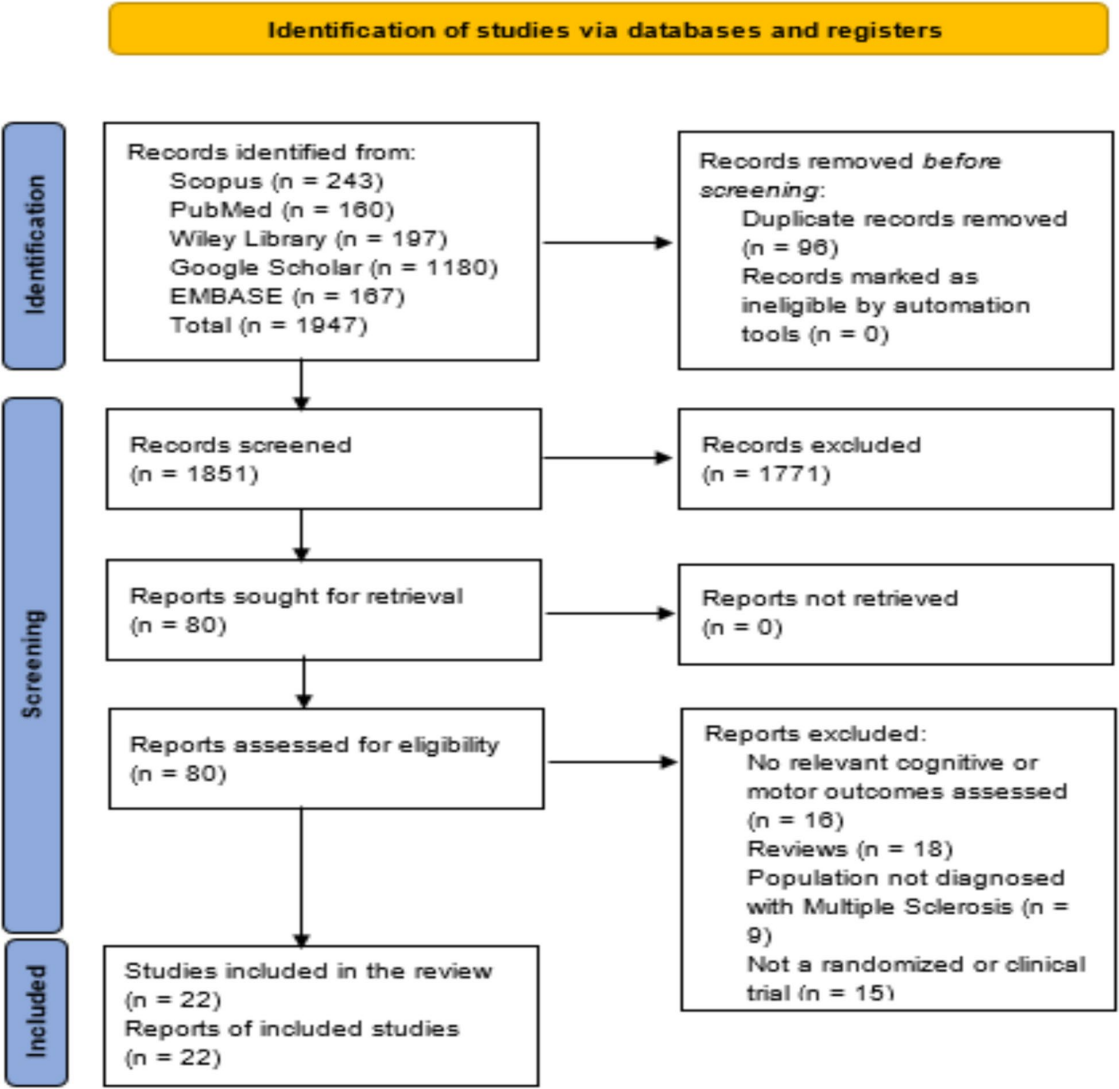
PRISMA flow diagram

The literature search yielded 1947 records, of which 96 duplicates were removed. Automation tools automatically retracted zero records. Further, 1771 articles were excluded following title and abstract screening. The remaining 80 articles were sought for retrieval, after which 22 studies that met the eligibility criteria were included. The results are presented in Fig. 1 in the PRISMA flow diagram [39]. Of the 22 included studies, 17 were full randomized controlled trials, 2 were pilot RCTs, one was quasi-experimental, and 2 used crossover designs.

### Data extraction results

This review included 22 studies (17 full RCTs, 2 pilot RCTs, 1 quasi-experimental study, and 2 crossover trials) published between 2015 and 2025, with sample sizes ranging from 5 to 120 participants. The studies involved adults with Multiple Sclerosis across various countries, including Iran, the United States, Italy, Germany, and Japan. Additionally, most studies investigated the effects of anodal tDCS over the motor cortex, DLPFC, or cerebellum, often combined with cognitive or physical training, and outcomes assessed included balance, gait, dexterity, cognitive function, and quality of life, as shown in the study characteristics Table 2 in the appendix.

**Table 2:** Study characteristics

Study (Design)	Sample size and characteristics and MS subtype	Intervention Parameters (Type of tDCS, Electrode placement, Intensity, Session duration/number)	Target Site	Outcome Measures	Key Results	Reported adverse events and dropout rates
Akbari et al. 2024 (RCT) [31]	51 MS patients; MS subtype not specified	Anodal tDCS (a-tDCS); Electrode placement: cerebellum or dorsolateral prefrontal cortex (DLPFC); Intensity: 1.5 mA; Session: 20 min, 10 sessions over 4 weeks; Sham: stimulation for 30 s then off	Cerebellum/DLPFC	Fatigue Severity Scale (FSS), Timed Up and Go (TUG) test, Berg Balance Score (BBS)	Significant reduction in fatigue with DLPFC a-tDCS + postural training (*P* < 0.001); Significant improvement in balance with cerebellar a-tDCS + postural training (*P* <0.001); No significant changes in sham (P > 0.001)	None reported
Baroni et al. 2022 (RCT) [30]	16 subjects with multiple Sclerosis (MS subtype not specified)	ctDCS (real or sham) combined with task-oriented training; applied daily for 2 weeks	Cerebellum	Functional mobility, balance, walking performance, quality of life, psychological and executive functions	Motor, QoL and mobility improved	Not reported
Charehjou et al. 2024 (RCT) [33]	30 MS patients, aged 18–55 years; EDSS ≤ 6; “brain type” MS	tDCS group: Anodal tDCS, anode on left M1 (C3), cathode on right forehead; 2 mA, 20 min daily for 5 consecutive days. VR group: VR BOX headset, 20 min per session, 3 sessions/week for 2 weeks	M1 motor cortex	BBS, T25-FW and FSS	All groups improved fatigue post-test. VR and tDCS + VR groups significantly improved balance and walking speed compared to tDCS alone (*P* <0.001)	No dropouts; all participants completed study. No adverse events reported
Charvet et al. 2017 (Clinical trial) (Clinical trial) [41]	45 adults with Multiple Sclerosis (MS). MS subtype not specified	RS-tDCS: 1.5 mA × 20 min, dorsolateral prefrontal cortex (DLPFC) montage. 10 sessions. Control: CT only (10 × 20 min)	DLPFC	BICAMS, attention tests	RS-tDCS + CT group showed significantly greater improvement in complex attention and response variability	Not reported
Charvet et al. 2023 (RCT) (RCT) [34]	60 progressive MS (32% primary, 68% secondary); right-hand dominant; ages 37–72; 52% female; median EDSS = 5.0 (range 1.5–7.5)	Active tDCS: 2.0 mA, anode over primary motor cortex (M1), cathode over supraorbital region (M1-SO montage); paired with daily manual dexterity training; 20 sessions (M–F) over 4 weeks	M1-SO	9HPT, MMPUT	Manual dexterity improved (p = 0.001), Active tDCS significantly improved 9HPT for both hands and trended towards improvement on MMPUT	Well tolerated, 98% completion and no adverse events were reported
Ehsani et al. 2022 (RCT) [32]	37 MS patients (MS subtype not specified)	Cerebellar a-tDCS: 1.5–2.0 mA (not explicitly stated, typical), 20 min, 10 sessions	Cerebellum	BBS, Biodex, FES-I	Cerebellar a-tDCSC + postural training group showed significant improvements in posture, balance, and fear of falling immediately and one-month post-intervention (*p* < 0.001)	Not reported
Fiene et al. 2018 (RCT) [42]	15 MS patients, MS subtype not specified	Anodal vs. Sham over the Left dorsolateral prefrontal cortex (DLPFC)	Left DLPFC	SRT, P300, fatigue	Anodal tDCS increased P300 amplitude (persisted post-stimulation) and prevented fatigue-related RT increase during testing session	Not reported
Gholami et al. 2021 (RCT) [40]	24 MS patients aged 18–40 years with an EDSS 1–4	Anodal vs. sham over the Left dorsolateral prefrontal cortex (DLPFC) at an intensity of 2 mA 20 min × 8 consecutive daily sessions	Left DLPFC	CBS-CP, RBANS	Real tDCS group showed significant improvements in reasoning and executive functions; attention improved but not significantly	Not reported
Grigorescu et al. 2020 (Crossover) [36]	11 MS patients; 10 relapsing–remitting MS, 1 secondary-progressive MS with a mean disease duration 75.6 months; mean EDSS = 3.14 ± 1.31	Anodal vs. sham tDCS over the left dorsolateral prefrontal cortex (DLPFC) at an intensity of 2 mA, 20 min × single session	Left DLPFC	N-Back, SDMT, social cognition	Accuracy on 1-Back test improved after sham but not after active bifrontal tDCS and no significant tDCS effects on SDMT, Eyes Test, or Faux Pas Test	Well tolerated and no serious adverse events were reported
Marotta et al. 2022 (RCT) [27]	17 RRMS patients with an EDSS > 1 and < 5	Anodal tDCS; M1–SO montage (anode over C3 contralateral to most-affected side, cathode over Fp2); 2.0 mA; 20 min; 10 sessions (5 days/week × 2 weeks)	Primary motor cortex (M1)	BBS, TUG, 6MWT	Significant short-term improvements in 6MWT distance (ΔT0–T1, p = 0.003), BBS (p = 0.03); gains not sustained at 4–6 weeks	No side effects
Mattioli et al. 2015 (RCT) [16]	20 right-handed patients with Relapsing–Remitting MS (RRMS), age 18–65, EDSS < 5	a-tDCS using battery-driven DC stimulator; anode: left DLPFC; cathode: extracephalic (right shoulder, 60 cm^2^, 0.03 mA/cm^2^). 20 min per session × 10 sessions (5 days/week for 2 weeks)	Left DLPFC	SDMT, WCST, PASAT	a-tDCS + training led to significantly greater improvements in SDMT and WCST	All patients completed treatment and no adverse rate or dropouts were reported
Mohammad et al. 2022 (RCT) [28]	29 MS adults patients, (MS subtype not specified)	A-tDCS delivered via ActivaDose; anode over M1, cathode over left supraorbital area; 2 mA; 20 min/session; once daily for 5 consecutive days	M1 cortex	BBS, TUG, 6MWT, QoL	significant improvements were observed in BBS, TUG, and 6MWT compared to baseline and sham (all *p* < 0.05)	No adverse events or dropouts were reported
Nguemeni et al. 2022 (RCT) [40]	22 MS patients with an EDSS 2–6.5	Anodal cerebellar tDCS vs sham with 6 sessions in total	Cerebellum	FGA, 2MWT	No significant tDCS vs sham difference was observed	No adverse events were reported
Pilloni et al. 2020 (RCT) [29]	17 RRMS and SPMS patients with an EDSS 1.0–6.5	2.5 mA, 20 min, single session	M1 (C3)	10—m walk, TUG	No significant changes in gait speed or TUG after a single session in either group	Stimulation was well tolerated and no dropouts were reported
Pilloni et al. 2020-2 (RCT) ]—2 (RCT) [26]	15 RRMS/SPMS patients with a median EDSS of 5.3	Anodal tDCS over M1 (C3 anode, Fp2 cathode); 2.5 mA; 20 min/session; 10 sessions (5 days/week × 2 weeks)	Primary motor cortex (M1, left hemisphere)	10MWT, 2MWT, gait parameters	Significant improvements in gait speed, stride length, and cadence at 10th session and maintained at 4-week follow-up	No tDCS-related adverse events were observed
Pilloni et al. 2024 (RCT) (RCT) [35]	65 right-hand dominant participants with progressive multiple Sclerosis (PMS) and hand impairment	Active or sham M1–SO tDCS paired with manual dexterity training; 20 sessions over 4 weeks	Primary motor cortex–supraorbital (M1–SO) montage	9-HPT, DMMPUT, QoL	Left-hand function and QoL improved	High adherence and completion rates were observed and no adverse events were reported
Rahimibarghani et al. 2022 (RCT) [25]	39 MS patients (subtypes not specified)	Anodal tDCS + stationary cycling vs sham tDCS + cycling	M1 (C3)	2MWT, 5MWT, TUG	Walking tests improved with exercise + tDCS	Not reported
Roozbeh et al. 2024 (RCT) [37]	120 MS patients (subtype not specified)	Not specified	Not specified	DTI, BDNF, SDMT, CPT	tDCS + cognitive rehab boosted BDNF, DTI metrics, and cognition	Not reported
Shibuya et al. 2025 (Crossover) [39]	5 patients (3 with MS: 2 RRMS, 1 SPMS; 2 with NMOSD); mean age 59.2 y; mean disease duration 22.8 y	Anodal tDCS with sponge electrodes, anode over left M1 (C3), cathode over Fp2; 1.0 mA for 900 s; 10 sessions over 2 weeks	Left primary motor cortex (M1)	PASAT, fMRI connectivity, working memory and information processing	Active tDCS resulted to significant improvement in working memory and information-processing ability	No adverse events
Simani et al. 2022 (RCT) [43]	80 RRMS patients with cognitive impairment	10 daily sessions	Not specified	Cognitive function	All cognitive domains improved vs sham	Not reported
Workman et al. 2019 (Crossover) [24]	12 patients with RRMS with a moderate disability PDDS 2–6 and unilateral leg weakness	Anodal tDCS; anode over M1 of more-affected leg (C3/C4), cathode on contralateral supraorbital area; 2 mA, 5 × 5 cm electrodes	Primary motor cortex (leg area, C3 or C4)	Walking speed, walking distance, step length	tDCS before walking improved walking speed and showed positive trends for distance and step length; tDCS during walking reduced distance and showed negative trends for speed and step length; timing significantly influenced outcomes	No discomfort from Tdcs placement were observed
Zakibakhsh et al. 2024 (RCT) [38]	40 MS patients (MS subtype not specified)	Anodal tDCS over left dorsolateral prefrontal cortex (F3), cathodal over right frontopolar cortex (Fp2); 1.5 mA; 20 min; 10 sessions	Left DLPFC	QoL, sleep, cognition	Active tDCS significantly improved quality of life, sleep quality, and reduced psychological distress vs sham; also improved psychomotor speed, attention, and vigilance	No serious adverse events were reported

### Methodological quality assessment

**Fig. 2:**
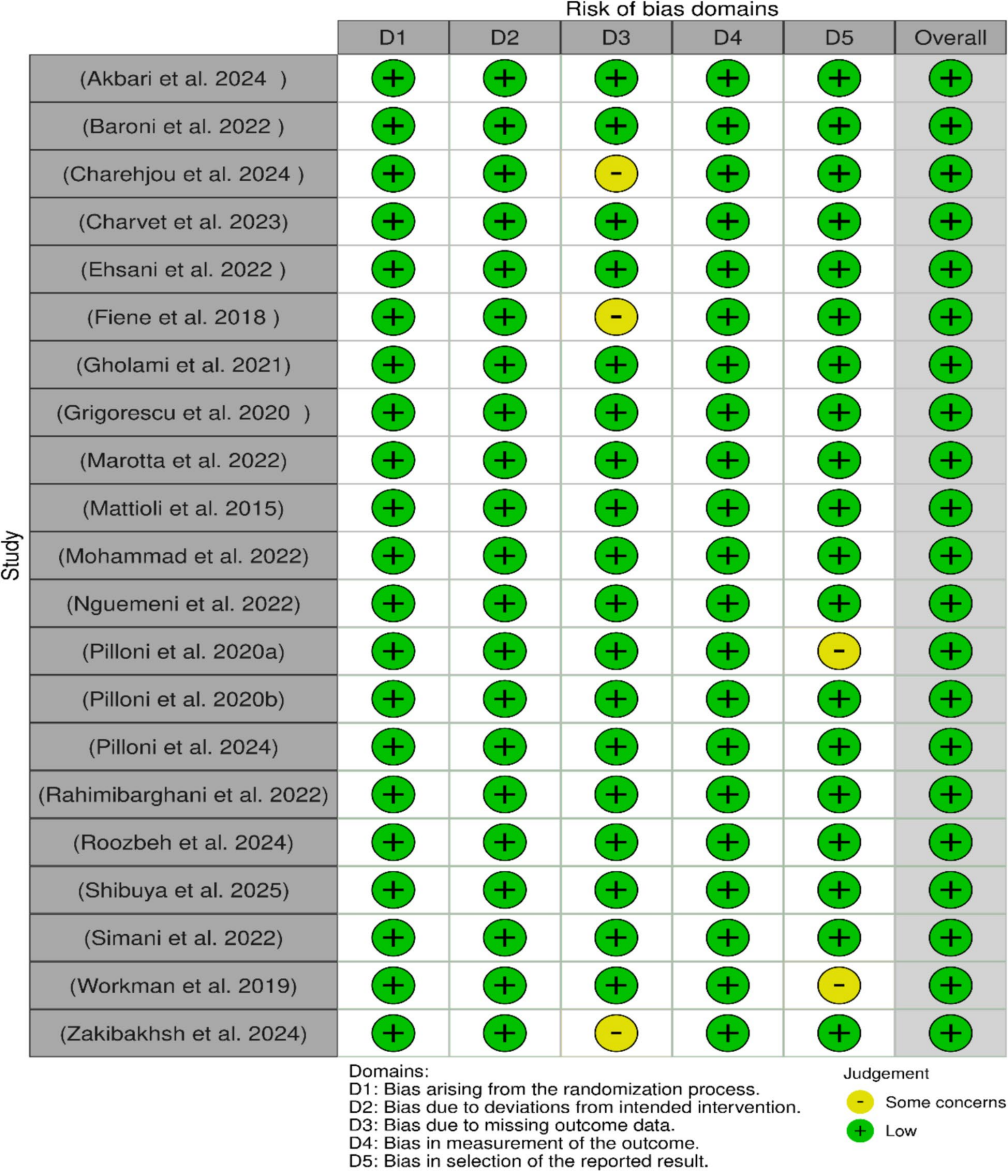
Traffic lights plot of Rob 2.0 risk of bias assessment results

**Fig. 3:**
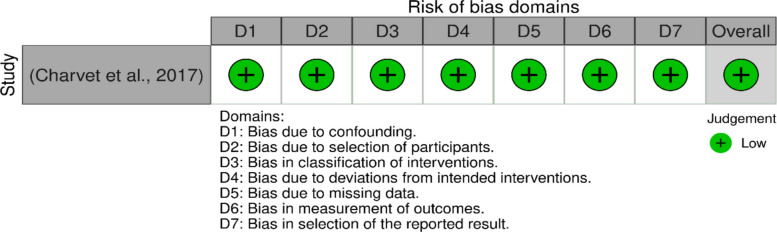
Traffic lights plot of Robins-I risk of bias assessment results

**Fig. 4:**
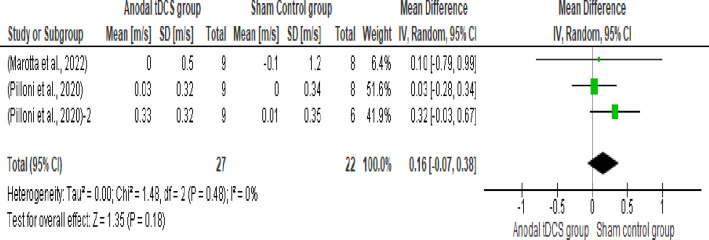
Forest plot of comparison of gait speed between the anodal tDCS intervention and the Sham control group

**Fig. 5:**
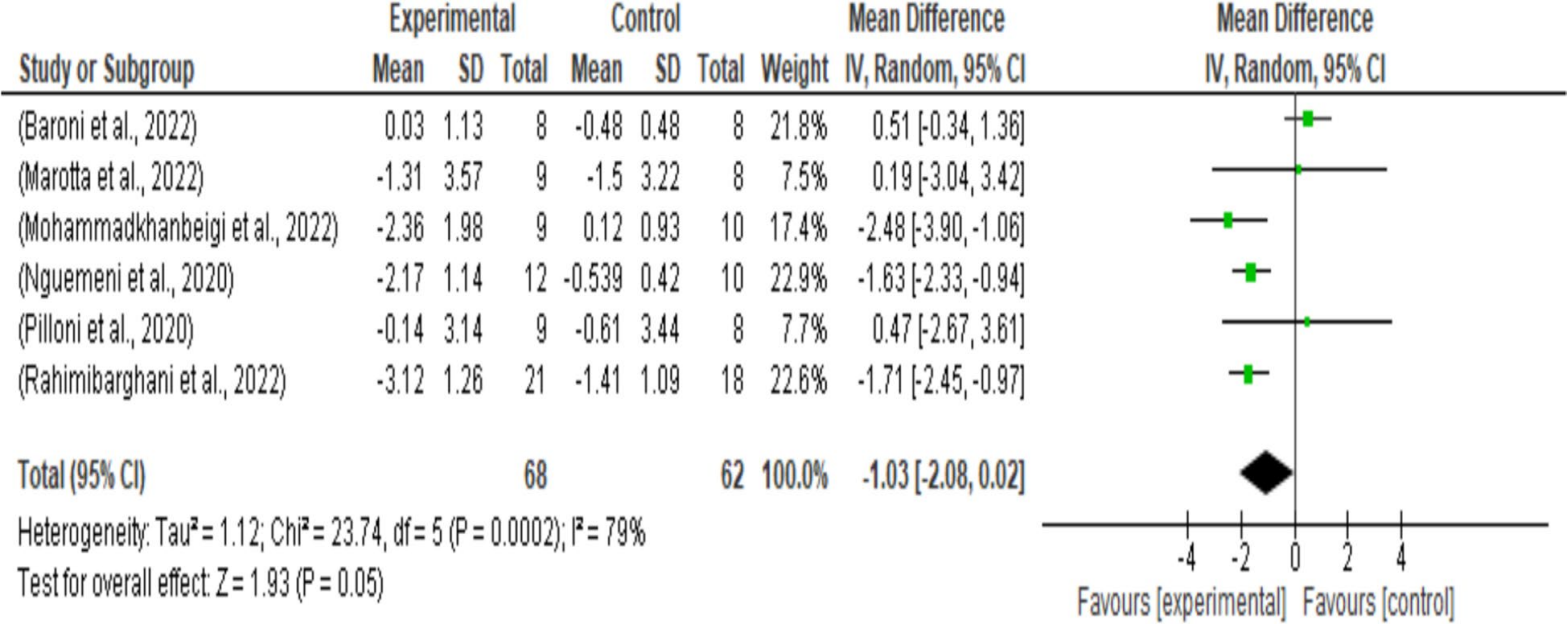
Forest plot comparing the TUG test between the tDCS and Sham intervention groups

The methodological quality assessment revealed predominantly low risk of bias across included studies, as shown in Figs. 2, 3, 4 and 5.

### Thematic analysis of outcomes

#### Efficacy of tDCS on motor functions in MS patients

##### Walking and gait performance

Multiple studies have demonstrated significant improvements in walking capacity and gait parameters following tDCS interventions, with the timing and application method of tDCS proving to be crucial for optimal outcomes. Research by [50] revealed that anodal tDCS applied before walking tasks (BEFORE group) in relapsing–remitting multiple sclerosis (RRMS) patients significantly increased gait velocity compared to sham stimulation (*p* = 0.04). In contrast, stimulation applied during walking (DURING group) significantly decreased the walking distance (*p* = 0.026), indicating that pre-activation of the motor cortex is more beneficial than concurrent stimulation. Several studies also reported improvements in walking capacity measures following anodal tDCS intervention, with a study by [43] revealing that exercise combined with anodal tDCS in PPMS, RRMS, and SPMS patients achieved significantly higher post-treatment values than exercise with sham tDCS in both 2-Minute Walk Test and 5-Meter Walk Test performance, with benefits maintained at 1-month follow-up. Similarly, (Pilloni, Choi, Coghe, et al., 2020) reported within-group improvements in gait speed (from 0.87 to 1.20 m/s) and 2-min walking test distance (from 118.53 ± 47.52 to 133.06 ± 49.2 m) in the active tDCS group,however, no statistically significant between-group differences versus sham were observed in that session, limiting the interpretation of these within-group changes as evidence of tDCS efficacy per se. Additionally, the gait speed remained elevated at 1.18 m/s (*p* < 0.001) and distance traveled further increased to 143.82 m (*p* < 0.001) compared to baseline. The anodal tDCS group demonstrated significant improvements in motor function compared to sham control, with statistically significant between-group differences in gait performance (6MWT: RBC = 0.8,*p* = 0.003) at the end of treatment (T1) [32]. At T1, the anodal tDCS group showed significant increases in walking distance with a mean difference of 37.0 m [− 59.0, 17.0] (*p* = 0.003). Additionally, the experimental group showed significant differences in repeated measures compared to control (*p* = 0.03, W = 0.34 versus *p* = 0.15, W = 0.20), with a substantial increase in gait cycle length (*p* = 0.04, W = 0.31) while the control group experienced significant decreases in gait velocity (*p* = 0.01, W = 0.77) [32].

The anodal tDCS group demonstrated significant improvements in balance and walking capacity post-intervention (*p* < 0.05), with effects comparable to those achieved through core stability training exercises [37]. Additionally, mobility was significantly improved in the tDCS group specifically (*p* < 0.05), indicating enhanced motor function outcomes. ANOVA analysis revealed no significant main effects of the intervention [F(1,15) = 0.074, *p* = 0.861, η2 = 0.006 for gait speed,F(1,15) = 0.087, *p* = 0.883, η2 = 0.002 for TUG time], no significant time effects [F(1,15) = 2.346, *p* = 0.070, η2 = 0.101 for gait speed; F(1,15) = 1.784, *p* = 0.239, η2 = 0.088 for TUG time] (Pilloni, Choi, Coghe, et al., 2020). In another study, both the experimental group receiving tDCS stimulation in patients with RRMS and the control group undergoing sham treatment maintained similar gait performance across sessions, with post-intervention gait velocity values of 0.8 ± 0.5 m/s for the experimental group and 0.8 ± 1.2 m/s for the control group at T2, indicating no significant improvement in gait velocity following the 10-session intervention [32].

RRMS patients who received tDCS intervention before providing a six-minute walk test demonstrated a gait speed of 1.03 ± 0.21 m/s, higher than in the sham group with 1.01 ± 0.23 m/s gait speed [50]. Similarly, both groups of RRMS and SPMS patients receiving tDCS intervention and the sham group maintained similar gait performance across sessions, with post-intervention gait speed values of 0.95 ± 0.32 m/s for the active group and 0.96 ± 0.34 m/s for the sham group (Pilloni, Choi, Coghe, et al., 2020). There were no statistically significant changes in gait speed from pre- to post-intervention in either group (*p* = 0.456 for the active group, *p* = 0.558 for the sham group), indicating no measurable improvement in walking speed following the intervention. In a different study, the active group treated with anodal tDCS showed a remarkable improvement in gait speed, where the result increased in speed from 0.87 ± 1.32 m/s in the baseline to 1.20 ± 0.32 m/s. By comparison, none of these changes were observed in the sham group with a gait speed of 0.95 ± 0.33 m/s at baseline and 0.96 ± 0.35 m/s on the 10th day [40, 41, 41].

Both the experimental group of RRMS patients receiving anodal tDCS and the control group undergoing sham treatment showed improvements in mobility, with post-intervention TUG values of 12.8 ± 5.0 s for the experimental group and 11.3 ± 3.9 s for the control group at T1 [32]. Similarly, both the active tDCS and sham groups of both RRMS and SPMS patients showed minimal changes in functional mobility as assessed by the Timed Up and Go (TUG) test, with post-intervention values of 14.34 ± 4.02 s for the active group and 14.58 ± 4.33 s for the sham group,however, these differences were not statistically significant (*p* = 0.195 and *p* = 0.103, respectively) (Pilloni, Choi, Coghe, et al., 2020). In another study, the anodal tDCS intervention showed meaningful changes in functional mobility, whereby the post-intervention TUG was 6.62 ± 1.81 s compared to 7.78 ± 1.30 s for the sham tDCS group and 6.78 ± 1.28 s for the core stability exercise group [37]. The result revealed that the anodal tDCS group significantly improved (*p* = 0.002), but the sham group showed no significant change (*p* = 0.476). Functional mobility, as measured by the TUG, in both RRMS, SPMS, and PPMS patients improved significantly in the anodal tDCS plus exercise group as compared to the exercise-only group, with the post-intervention TUG value of the former being 12.72 ± 1.05 s as opposed to values of 13.52 ± 1.16 s of the latter [43]. The anodal tDCS plus exercise group showed a statistically significant improvement (*p* = 0.001) with a relative change of −16.9%, while the exercise-only group also demonstrated significant improvement (*p* = 0.002) but with a more minor relative change of −8.5%. The results of another study were the same, where the real Cerebellar tDCS plus Task-oriented circuit training (TOCT) intervention in multiple sclerosis patients exhibited a marginal change of 0.03 ± 1.13 s on the TUG test at T1, which equated to practically no functional mobility improvement from baseline [5]. In contrast, the sham-ctDCS plus TOCT group showed slight improvement, decreasing −0.48 ± 0.48 s at T1, suggesting marginally better functional mobility performance.

##### Balance and postural control

Balance improvements represent another consistent finding across tDCS studies in MS patients. Cerebellar stimulation has shown particular promise in this domain, with research by [1] demonstrating that anodal tDCS over the cerebellum combined with postural training produced marked improvements in Berg Balance Scale and Timed Up and Go test scores compared to both dorsolateral prefrontal cortex stimulation and sham groups. Similarly, Cerebellar anodal tDCS (a-tDCS) combined with postural training significantly improved motor outcomes related to posture and balance in patients with MS [13]. Improvements were observed in Postural Stability Indices and balance scores. In contrast, control groups (sham and postural training alone) showed only short-term improvements (*p* < 0.05), which were not maintained after one month (*p* < 0.05) [13]. VR and combined tDCS-VR interventions showed greater improvements in motor functions, particularly in balance (measured by the Berg Balance Scale) and walking speed (measured by the 25-foot walk test),however, the tDCS-VR group did not show significantly greater improvements than the VR-only group [8].

The anodal tDCS group achieved a post-intervention Berg Balance Scale score of 39.33 ± 4.71, representing a statistically significant improvement (*p* < 0.001) of 5.33 points from baseline. In comparison, the sham tDCS group showed no change with identical pre- and post-test scores (41.7 ± 6.25) [37]. Similarly, the experimental group receiving anodal tDCS significantly improved balance performance, with BBS scores increasing from 48.3 ± 7.4 at T0 to 53.3 ± 3.9 at T1 (*p* = 0.023) [32]. In contrast, the control group slightly increased from 50.4 ± 1.4 to 51.4 ± 4.4 [32]. In the group receiving cerebellar a-tDCS, post-intervention Berg Balance Scale exhibited final values of 8.25 ± 4.07, while the sham tDCS group showed a final Berg Balance Scale of 4.75 ± 6.14 [13].

##### Manual dexterity and fine motor skills

There was a significant improvement in manual dexterity among individuals with progressive multiple Sclerosis who received active transcranial direct current stimulation (tDCS) over the primary motor cortex (M1-SO) alongside daily home-based dexterity training, with the Nine-Hole Peg Test (9HPT) showing marked improvements in both right-hand (*p* = 0.036) and left-hand (*p* = 0.028) impaired participants compared to the sham stimulation group [9]. The overall group that received training showed a modest improvement (mean z-score improvement 1.64 ± 9.53, *p* = 0.016),however, participants receiving active tDCS demonstrated a significantly higher mean z-score improvement of 4.51 ± 8.78 (*p* = 0.001), indicating that the addition of tDCS substantially enhanced motor rehabilitation outcomes. In another study, the progressive MS (PMS) patients who received active tDCS demonstrated significant improvements in manual dexterity compared to baseline, with the left hand showing significant enhancement in Nine-Hole Peg Test performance (− 5.85 ± 6.19 vs − 4.23 ± 4.34, *p* = 0.049) and Dellon-Modified-Moberg-Pick-Up Test scores (− 10.62 ± 8.46 vs − 8.97 ± 6.18, *p* = 0.049) compared to the sham group [42].

#### Efficacy of tDCS on cognitive functions in MS patients

##### Processing speed and attention

Among the well-known areas of tDCS effectiveness in MS patients is cognitive improvement, where both processing speed and attention have been identified to be steadily improved in different studies. Processing speed is an essential part of therapeutic interventions, and considerable gains have been made in a critical measure of processing speed, the Symbol Digit Modalities Test (SDMT). Research by Mattioli et al. [33] demonstrated a significant increase in the SDMT due to anodal tDCS over the left dorsolateral prefrontal region using anodal stimulation, and the effects persisted at the six-month point of assessment. In this case, the anodal tDCS group improved significantly (*p* = 0.019) in information processing speed with + 8.8 ± 8.6 points on the SDMT. In contrast, the sham group showed no difference of 0.1 ± 6.7 points [33]. In another research, the active tDCS stimulation group improved in the speed of information processing, non-significantly (*p* = 0.083), with an increment of + 4.27 ± 18.68 in the experimental group compared to 1.9 ± 15.46 in the control group [19]. On the same note, a combination of tDCS and application-based cognitive rehabilitation group showed significantly higher concentration of BDNF levels. Additionally, the intervention increased fractional anisotropy scaled down DTI over other groups of treatment and significant improvement in cognitive capability in a variety of areas such as processing speed (SDMT), attention (IVA-CPT), and executive working memory (IGT) [45].

In the active tDCS group, a significant difference in cognitive outcomes was seen when compared with the sham tDCS group, as repeated prefrontal stimulation aided in the improved psychomotor speed (*p* < 0.05), attention (*p* < 0.05), and vigilance (*p* < 0.05) [51]. The test of cognitive enhancements was significant and positively correlated with improvements in mental health indicators, including psychological distress and sleep quality.

##### Working memory and executive function

Working memory and executive function domains have shown robust improvements with tDCS interventions. The active tDCS group demonstrated significant improvement in working memory and information-processing ability as assessed by the Paced Auditory Serial Addition 2—s version compared to sham stimulation, with anodal tDCS combined with rehabilitation showing measurable cognitive enhancement in MS patients [47]. Similarly, Anodal transcranial direct current stimulation (tDCS) over the left dorsolateral prefrontal cortex (DLPFC) for eight consecutive sessions resulted in significant improvements in reasoning and executive function in MS patients compared to sham stimulation. At the same time, attention also showed notable improvement, though it was not statistically significant [16]. Another study by Mattioli et al. [33] provided comprehensive evidence for executive function improvements following anodal tDCS in multiple sclerosis patients, showing substantial enhancements in Wisconsin Card Sorting Test performance, including reductions in total errors, perseverative responses, perseverative errors, and non-perseverative errors, with many benefits sustained at six-month follow-up.

##### Complex attention and sustained performance

Complex attention tasks have shown particular responsiveness to tDCS interventions. Remotely-supervised transcranial direct current stimulation (RS-tDCS) and cognitive training significantly improved complex attention (*p* = 0.01). It reduced intra-individual response variability (*p* = 0.01) compared to cognitive training alone. At the same time, no significant differences were found in basic attention (*p* = 0.95) or standard cognitive tests (BICAMS composite score, *p* = 0.99) after 10 sessions [10]. Similarly, Fiene et al. [15] provided neurophysiological evidence for attention improvements, showing that anodal tDCS over the left dorsolateral prefrontal cortex significantly increased P300 amplitude during auditory tasks and prevented fatigue-related increases in reaction time, indicating enhanced sustained attention and alertness in MS patients experiencing cognitive fatigue. In another study, Significant improvements in Fall Efficacy Scale-International (FES-I scores (*p* < 0.001) were seen only in the cerebellar a-tDCS-PT group, indicating that cerebellar a-tDCS may modulate cognitive-emotional aspects such as fear-related self-efficacy [13]. In contrast, the control groups showed no significant change in FES-I (*p* < 0.05), indicating that postural training alone may not influence this cognitive parameter.

##### Comprehensive cognitive enhancement

Some studies have demonstrated broad cognitive improvements across multiple domains. A study by Simani et al. [48] found that all cognitive functions tested in multiple sclerosis patients, such as episodic memory, attention, inhibitory control, working memory, and visuospatial skill, significantly improved after ten consecutive daily sessions of anodal tDCS, with effects persisting at 4-week and 12-week follow-up assessments.

#### Safety and tolerability profile

The safety profile of tDCS in MS patients was generally favorable across reviewed studies. It should be noted that not all included studies formally reported adverse events, and safety reporting standards varied; therefore, the following summary reflects only those studies that explicitly addressed tolerability. Among reporting studies, intervention involving remotely supervised transcranial direct current stimulation (RS-tDCS) paired with at-home dexterity training was found to be safe, well tolerated, and highly feasible among individuals with progressive MS, with 98% of participants in that study completing at least 18 of the 20 prescribed sessions [9]. All participants tolerated the tDCS intervention similarly, with 2.5 mA stimulation level tolerated by all participants when given the 90-s tolerability test as the baseline (Pilloni, Choi, Coghe, et al., 2020). No serious adverse events were reported in any of the included studies. Minor, transient side effects such as tingling, itching, and warmth were mentioned in some studies,none of the reported side effects exceeded level 7 on a 0–10-point discomfort scale, and none prompted session or treatment discontinuation. In another study, there were no adverse events across 10 sessions of active tDCS in five patients with multiple sclerosis, showing a satisfactory safety and tolerability profile [47].

### Sub-group analysis

#### Motor function outcomes across MS subtypes

##### Relapsing–Remitting Multiple Sclerosis (RRMS)

Relapsing–Remitting Multiple Sclerosis (RRMS) patients demonstrated the most consistent and robust responses to tDCS interventions across motor function domains. RRMS patients showed significant improvements in walking and gait performance when tDCS was applied before walking tasks. Research by Workman et al. [50] reported significantly increased gait velocity compared to sham stimulation (*p* = 0.04). The timing of stimulation proved crucial, as pre-activation of the motor cortex yielded superior outcomes compared to concurrent stimulation during walking. Balance and postural control also showed significant improvements in RRMS patients, with Berg Balance Scale scores increasing from 48.3 ± 7.4 at baseline to 53.3 ± 3.9 post-intervention (*p* = 0.023), as Marotta et al. [32] reported. Additionally, the experimental group of RRMS patients receiving anodal tDCS demonstrated superior functional mobility outcomes compared to sham controls, with post-intervention TUG values of 12.8 ± 5.0 s for the experimental group versus 11.3 ± 3.9 s for controls.

##### Combined RRMS and SPMS patient populations

Several studies included mixed populations of both RRMS and SPMS patients, making it challenging to distinguish subtype-specific responses. Pilloni et al. (40, 41 examined combined RRMS and SPMS populations. They demonstrated remarkable gait speed improvements from 0.87 ± 1.32 m/s at baseline to 1.20 ± 0.32 m/s post-intervention, with enhancements in 2-min walking test distance from 118.53 ± 47.52 to 133.06 ± 49.2 m, and benefits maintained at 4-week follow-up. However, these studies did not provide separate analyses for RRMS versus SPMS patients, limiting the ability to determine differential treatment responses between these subtypes. The combined populations showed that while improvements were achievable, the heterogeneity in disease progression and symptom severity between RRMS and SPMS patients may have contributed to the variable responses observed across different outcome measures.

##### Secondary Progressive Multiple Sclerosis (SPMS)

Limited studies specifically examined SPMS patients as a distinct subgroup, making it challenging to draw definitive conclusions about treatment efficacy in this population. The evidence suggests that SPMS patients may show more variable responses to tDCS interventions, potentially requiring different stimulation protocols or extended intervention periods to achieve optimal outcomes.

##### Primary Progressive Multiple Sclerosis (PPMS)

PPMS patients represented the smallest subgroup in the analyzed studies, limiting the ability to draw definitive conclusions about treatment efficacy. The evidence from [43] suggested that PPMS patients could significantly improve when tDCS was combined with exercise interventions. The study reported that exercise combined with anodal tDCS in PPMS patients achieved substantially higher post-treatment values than sham tDCS in both 2-min and 5-m Walk Test performance, with benefits maintained at 1-month follow-up.

##### Progressive Multiple Sclerosis (PMS)

Progressive MS patients, including both SPMS and PPMS, showed significant improvements in manual dexterity following tDCS interventions. Study by [9] focusing on progressive MS patients revealed substantial improvements in manual dexterity among individuals who received active tDCS over the primary motor cortex (M1-SO) alongside daily home-based dexterity training, with the Nine-Hole Peg Test (9HPT) showing marked improvements in both right-hand (*p* = 0.036) and left-hand (*p* = 0.028) impaired participants compared to sham stimulation. The active tDCS group demonstrated significantly higher mean z-score improvement of 4.51 ± 8.78 (*p* = 0.001), indicating that tDCS substantially enhanced motor rehabilitation outcomes. Similarly, [42] reported that progressive MS patients receiving active tDCS demonstrated significant improvements in manual dexterity compared to baseline, with the left hand showing significant enhancement in Nine-Hole Peg Test performance (− 5.85 ± 6.19 vs − 4.23 ± 4.34, *p* = 0.049) and Dellon-Modified-Moberg-Pick-Up Test scores (− 10.62 ± 8.46 vs − 8.97 ± 6.18, *p* = 0.049) compared to the sham group. These findings suggest that progressive MS patients may retain significant potential for motor learning and rehabilitation in fine motor skills despite disease progression.

#### Cognitive function outcomes across MS subtypes

##### Cognitive outcomes in mixed MS populations

Most cognitive studies included mixed MS populations without providing subtype-specific analysis, making it challenging to determine differential cognitive responses between MS subtypes. A study by Mattioli et al. [33] demonstrated significant improvements in processing speed with anodal tDCS over the left dorsolateral prefrontal cortex, showing substantial increases in Symbol Digit Modalities Test (SDMT) scores, with the anodal tDCS group improving by + 8.8 ± 8.6 points (*p* = 0.019) compared to no difference in the sham group. Working memory and executive function improvements were also reported, with significant enhancements in Wisconsin Card Sorting Test performance, including reductions in total errors, perseverative responses, and perseverative errors, with benefits sustained at six-month follow-up [33]. However, these studies did not specify MS subtypes, limiting the ability to determine which patient populations may be most responsive to cognitive interventions.

#### Stimulation site-specific efficacy

##### Primary Motor Cortex (M1) Stimulation

Anodal tDCS over the primary motor cortex demonstrated the most consistent and robust effects on motor function outcomes across multiple studies. M1 stimulation showed particular efficacy for gait and walking performance, with numerous studies reporting significant improvements in gait velocity, walking distance, and functional mobility. The research by [40, 41, 41] demonstrated that M1 stimulation produced remarkable gait speed improvements from 0.87 ± 1.32 m/s at baseline to 1.20 ± 0.32 m/s post-intervention. Manual dexterity improvements were particularly pronounced with M1 stimulation, as evidenced by Charvet et al. [9], who reported significant improvements in Nine-Hole Peg Test performance in both right-hand (*p* = 0.036) and left-hand (*p* = 0.028) impaired participants compared to sham stimulation. The M1-SO (primary motor cortex—shoulder) configuration showed marked effectiveness when combined with daily home-based dexterity training, with active tDCS demonstrating significantly higher mean z-score improvement of 4.51 ± 8.78 (*p* = 0.001) compared to sham stimulation.

##### Dorsolateral Prefrontal Cortex (DLPFC) Stimulation

Left DLPFC stimulation emerged as the most effective site for cognitive enhancement in MS patients. Studies consistently reported significant improvements in processing speed, working memory, and executive function following DLPFC stimulation. A study by Mattioli et al. [33] demonstrated that anodal tDCS over the left DLPFC resulted in significant increases in SDMT scores, with the anodal tDCS group improving by + 8.8 ± 8.6 points (*p* = 0.019) compared to no difference in the sham group. In another study, the neurophysiological evidence provided by Fiene et al. [15] showed that DLPFC stimulation significantly increased P300 amplitude during auditory tasks and prevented fatigue-related increases in reaction time, indicating enhanced sustained attention and alertness.

##### Cerebellar stimulation

Cerebellar tDCS showed specialized efficacy for balance and postural control functions, with several studies demonstrating superior outcomes to other stimulation sites. Research by [1] reported that anodal tDCS over the cerebellum and postural training produced marked improvements in Berg Balance Scale and Timed Up and Go test scores compared to DLPFC stimulation and sham groups. The cerebellar stimulation approach showed particular promise for postural stability. Research by [13] demonstrated significant improvements in Postural Stability Indices and balance scores maintained at one-month follow-up, while control groups showed only short-term improvements. Interestingly, cerebellar stimulation also influenced cognitive-emotional aspects, with significant improvements in Fall Efficacy Scale-International (FES-I) scores (*p* < 0.001) observed only in the cerebellar a-tDCS-PT group, indicating modulation of fear-related self-efficacy [13].

#### Combined interventions

##### TDCS plus exercise training

The combination of tDCS with exercise interventions demonstrated superior outcomes compared to either intervention alone. A study by Rahimibarghani et al. [43] reported that exercise combined with anodal tDCS achieved significantly higher post-treatment values than sham tDCS in both 2-min and 5-m Walk Test performance across all MS subtypes, with benefits maintained at 1-month follow-up. The anodal tDCS plus exercise group showed statistically significant improvement (*p* = 0.001) with a relative change of −16.9% in TUG performance, while the exercise-only group demonstrated significant but more minor improvement (*p* = 0.002) with a relative change of −8.5%. This synergistic effect suggests that combining tDCS with physical rehabilitation may optimize treatment outcomes.

##### TDCS plus cognitive training

The combination of tDCS with cognitive training showed particular efficacy for cognitive rehabilitation in MS patients. Remotely-supervised tDCS combined with cognitive training significantly improved complex attention (*p* = 0.01) and reduced intra-individual response variability (*p* = 0.01) compared to cognitive training alone [10]. The research by [45] demonstrated that tDCS combined with application-based cognitive rehabilitation resulted in significantly higher BDNF levels and improved fractional anisotropy in DTI imaging, alongside significant improvements in processing speed (SDMT), attention (IVA-CPT), and executive working memory (IGT). These findings suggest that combined interventions may produce neuroplastic changes that enhance cognitive rehabilitation outcomes beyond what either intervention could achieve independently.

### Meta-analysis results

#### Gait speed

A meta-analysis of three studies comparing gait speed among patients with relapsing–remitting MS (RRMS) and secondary progressive MS (SPMS) who received anodal tDCS was conducted, as illustrated in the forest plot below. The studies showed mixed results with mean differences ranging from 0.03 to 0.32, indicating variable effects of anodal tDCS treatment. Two of the three studies demonstrated positive effects favoring tDCS [40, 41, 41, 42], while one study showed an adverse effect [32]. However, none of the studies show statistically significant results based on their confidence intervals crossing zero. The pooled result shows a mean difference of 0.16 (95% CI: −0.07, 0.38) favoring anodal tDCS intervention, but this overall effect was not statistically significant (*p* = 0.18), suggesting that anodal tDCS does not significantly improve the measured outcome compared to sham control. The heterogeneity analysis revealed no significant heterogeneity between studies (Tau^2^ = 0.00, Chi^2^ = 1.48, df = 2, I^2^ = 0%, *p* = 0.48), indicating that the results across studies were consistent and the variation in effect sizes was likely due to chance rather than actual differences between studies. A fixed-effects model was used in this forest plot due to the low heterogeneity, as indicated by an I^2^ value of 0% in Fig. 4.

#### Functional mobility (Timed Up and Go Test)

Six studies that included quantitative data for the TUG test were meta-analysed as shown in the forest plot in Fig. 4 below. The studies showed mixed results with mean differences ranging from 0.51 to −2.48 s, indicating variable effects on functional mobility improvement with tDCS treatment. The pooled result shows a mean difference of −1.03 s (95% CI: −2.08, 0.02) favouring tDCS intervention, and this overall effect was approaching but not statistically significant (*p* = 0.05), suggesting that tDCS may improve functional mobility as measured by the TUG test compared to sham control. The heterogeneity analysis revealed substantial heterogeneity among the studies (I^2^ = 79%, Chi^2^ = 23.74, df = 5, *p* = 0.0002), indicating considerable variability in the treatment effects across the included studies, as shown in Fig. 5. A random-effects model was used due to this high heterogeneity. To explore potential sources of this heterogeneity, we conducted post-hoc descriptive subgroup comparisons stratifying studies by stimulation site (M1 vs. cerebellar montage) and by co-intervention type (tDCS alone vs. tDCS combined with exercise). Studies combining tDCS with exercise tended to show greater TUG improvements (e.g., [43]: MD = − 1.12 s, [37]: MD = − 2.48 s) compared to studies using tDCS without exercise (e.g., [32]: MD = + 0.51 s). Similarly, single-session protocols (e.g., [40, 41, 41], Coghe) showed minimal change, whereas multi-session protocols showed greater effects. However, formal subgroup meta-regressions were not feasible due to the small number of studies (n = 6), which is insufficient to draw definitive conclusions about the sources of heterogeneity. This high heterogeneity substantially limits confidence in the pooled TUG estimate and should be interpreted accordingly.

#### Balance (Berg Balance Scale)

**Fig. 6:**
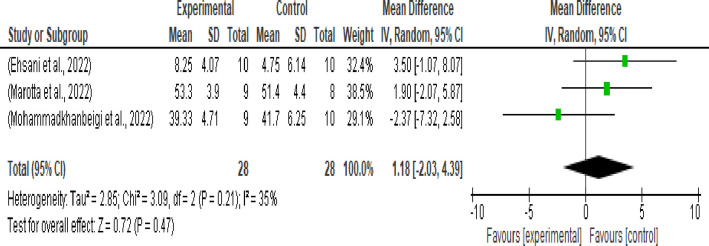
Forest plot comparison of BBS scores between the tDCS and Sham intervention groups

Three studies that included quantitative data for the Berg Balance Scale were meta-analyzed as shown in the forest plot in Fig. 6. The studies showed mixed results with mean differences ranging from −2.37 to 3.50, indicating variable effects on balance improvement with tDCS treatment. Two of the three studies demonstrated positive mean differences favouring the experimental group, while one showed a negative mean difference favouring the control group. The pooled result shows a mean difference of 1.18 (95% CI: −2.03, 4.39) favouring tDCS intervention, but this overall effect was not statistically significant (*p* = 0.47), suggesting that tDCS does not significantly improve balance as measured by the Berg Balance Scale compared to sham control. The analysis revealed moderate heterogeneity between studies (I^2^ = 35%, *p* = 0.21). While the heterogeneity test was not statistically significant, the I^2^ value of 35% indicates moderate heterogeneity according to Cochrane guidelines, suggesting some variability in treatment effects. A random-effects model was used in this forest plot due to moderate heterogeneity, as indicated by an I^2^ value of 35%. This is shown in Fig. 6.

#### Information processing speed (Symbol Digit Modalities Test)

**Fig. 7:**
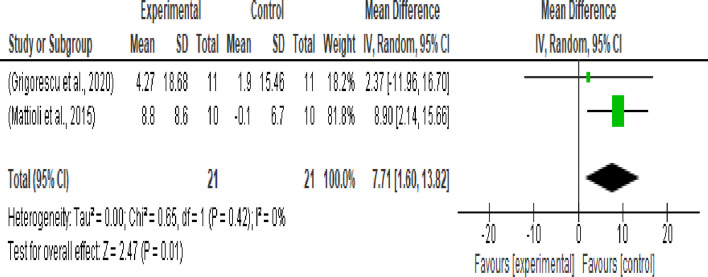
Forest plot of comparison of SDMT between the tDCS intervention and the Sham intervention group

Meta-analysis of two studies containing quantitative SDMT values was included in the forest plot as presented in Fig. 7 below. The studies showed consistent positive results with mean differences of 2.37 and 8.90, indicating improved information processing speed with tDCS treatment compared to control groups. Both studies demonstrate positive mean differences favouring the experimental group. The pooled result shows a mean difference of 7.71 (95% CI: 1.60, 13.82) favouring tDCS intervention, and this overall effect was statistically significant (*p* = 0.01), suggesting that tDCS significantly improves information processing speed as measured by the SDMT compared to sham control. Additionally, the analysis showed no heterogeneity between studies (I^2^ = 0%, *p* = 0.42), indicating consistent findings across the different studies included. A fixed-effects model was used in this forest plot due to the low heterogeneity, as noted in an I^2^ value of 0%. In the case of the funnel plot, with only two studies included in the SDMT analysis, meaningful assessment of publication bias through funnel plot analysis was not feasible. Publication bias assessment requires substantially more studies to provide a reliable interpretation, and these findings should be interpreted within this limitation.

#### Overall meta-analysis limitations

All meta-analyses were constrained by small study numbers (2—6 studies), substantially limiting statistical power and the reliability of pooled estimates. This represents a critical limitation in drawing definitive conclusions about tDCS efficacy. Most studies included mixed MS populations without providing subtype-specific analyses, preventing appropriate subgroup meta-analyses to determine differential treatment responses between RRMS, SPMS, and PPMS patients. Additionally, meaningful assessment of publication bias was not possible due to insufficient study numbers across all outcomes, representing a significant limitation in interpreting these results. A summary of the pooled meta-analysis results across all assessed outcomes is presented in Table 3 below.

**Table 3:** Summary table for meta-analysis results

Outcome Measure	No. of Studies	Pooled Mean Difference (95% CI)	*p*-value	Heterogeneity (I^2^, p)	Model Used	Interpretation
Gait Speed	3	0.16 (− 0.07, 0.38)	0.18	I^2^ = 0%, *p* = 0.48	Fixed-effects	No significant improvement
Functional Mobility (TUG)	6	− 1.03 (− 2.08, 0.02)	0.05	I^2^ = 79%, *p* = 0.0002	Random-effects	Trend toward improvement, not significant
Balance (BBS)	3	1.18 (− 2.03, 4.39)	0.47	I^2^ = 35%, *p* = 0.21	Random-effects	No significant improvement
Information Processing Speed (SDMT)	2	7.71 (1.60, 13.82)	0.01	I^2^ = 0%, *p* = 0.42	Fixed-effects	Significant improvement

##### GRADE quality evaluation

The high heterogeneity observed in functional mobility outcomes (I^2^ = 79%) and the diverse measurement tools used in assessing motor and cognitive functions compromised the certainty of evidence for some tDCS interventions. The limited number of studies for specific outcomes and potential publication bias further affected the quality of evidence. According to GRADE methodology, the certainty of evidence was downgraded from “High” for all pooled outcomes due to serious imprecision (very small numbers of studies, 2–6 per outcome) and, for the TUG outcome, also due to inconsistency (I^2^ = 79%). The SDMT outcome, based on only two small studies, is rated as Moderate certainty despite low heterogeneity, as two studies are insufficient to establish robust evidence. The TUG outcome is rated as Moderate certainty given inconsistency and imprecision. Gait speed and Berg Balance Scale outcomes are rated as Low certainty given the very small number of contributing studies and lack of statistical significance. Narratively synthesized outcomes (manual dexterity, working memory, executive function, complex attention) are rated as Low certainty due to inability to pool data and reliance on small, heterogeneous primary studies. The updated GRADE ratings are presented in Table 4 below.

**Table 4:** GRADE summary of findings for tDCS interventions in MS patients

Outcome	No. of Studies	Effect Size	Quality of Evidence
Gait Speed	3	0.16; 95% [CI], −0.07 to 0.38	Low
Functional Mobility (TUG)	6	−1.03; 95% [CI], −2.08 to 0.02	Moderate ↓
Balance (Berg Balance Scale)	3	1.18; 95% [CI], −2.03 to 4.39	Low
Information Processing Speed (SDMT)	2	7.71; 95% [CI], 1.60 to 13.82	Moderate ↓
Manual Dexterity	2	Not applicable	Moderate
Working Memory and Executive Function	3	Not applicable	Moderate
Complex Attention	2	Not applicable	Moderate

## Discussion

This study investigated the therapeutic potential of transcranial direct current stimulation (tDCS) in improving motor and cognitive functions in patients with multiple Sclerosis. Additionally, it evaluated the safety profile and clinical feasibility of tDCS intervention. The findings demonstrate several patterns in how tDCS affects motor and cognitive outcomes in MS patients, though the strength of evidence varies considerably across outcomes. The improvement in SDMT scores between tDCS and control groups (mean difference: 7.71 points, p = 0.01) represents a potentially meaningful cognitive enhancement in information processing speed. Significantly, this difference exceeds the SDMT's reported standard error of measurement of 4.38 points in MS patients, underscoring the reliability of the observed effect [31]. Understanding whether this change is clinically meaningful requires comparison against established MCID thresholds. Research by Jensen et al. [25] reports a distribution-based MCID estimate for the SDMT of 17.1%, which corresponds to approximately 7–8 points in the typical scoring range. Although this suggests that the observed 7.71-point improvement is close to what might be considered clinically meaningful, no universal MCID for the SDMT in MS has been universally validated. While our findings are promising, they are based on only two small studies, which limits confidence in the robustness of this effect despite low heterogeneity (I^2^ = 0%) [19, 33]. While processing speed deficits are among the most common and debilitating cognitive symptoms in MS patients [3], the preliminary nature of this evidence necessitates cautious interpretation until replicated in larger trials. This finding aligns with the direction of effects reported in previous meta-analyses by Hsu et al. [23] in neurological populations, though methodological differences limit direct comparisons. Similarly, the improvement in functional mobility in tDCS-treated patients (TUG mean difference: −1.03 s, *p* = 0.05) suggests enhanced practical motor performance relevant to daily activities [32, 43]. However, this result should be interpreted cautiously, given the borderline statistical significance and the small number of contributing studies. Additionally, the trend toward improved gait speed observed in individual studies suggests potential compensatory mechanisms through enhanced corticospinal excitability [40, 41, 41, 50], though pooled analysis of three studies failed to reach statistical significance (*p* = 0.18), indicating insufficient evidence for definitive conclusions. These findings complement the recent review by Lefaucheur et al. [30], which reported modest but consistent motor improvements with tDCS across neurological conditions. However, our findings show more pronounced effects specifically in MS populations.

The high prevalence of cognitive impairment in MS patients (40—65% even in early stages) represents a substantial clinical challenge, considering the limited pharmacological options available [3]. The preliminary improvements observed in working memory and executive function among tDCS-treated patients suggest potential benefits beyond acute stimulation effects [16, 47], though these findings require validation in larger, more rigorous trials. While motor outcome heterogeneity was observed across studies, the trend toward improvement in manual dexterity (Nine-Hole Peg Test improvements: *p* = 0.036 for right hand, *p* = 0.028 for left hand) warrants further investigation given the functional importance of upper limb coordination in MS patients [9, 42]. The observed improvements in complex attention and sustained performance suggest that tDCS may potentially enhance cognitive reserve mechanisms [10, 15], though the evidence base remains limited. This preliminary pattern contrasts with meta-analysis findings by Razza et al. [44] on tDCS in depression, where cognitive improvements were less pronounced, potentially suggesting disease-specific mechanisms in MS populations. However, this hypothesis requires further investigation.

The mechanisms underlying potential tDCS efficacy in MS likely involve multiple pathways. Anodal stimulation increases neuronal excitability and promotes long-term potentiation, potentially compensating for reduced cortical excitability due to demyelination and axonal damage [26, 28]. Moreover, the effects on cognitive and motor outcomes could be explained by augmented neuroplasticity due to regulating GABA and glutamate neurotransmission, leading to strengthening of synapses and facilitation of cortical reorganization [46]. These processes correlate with neuroimaging results indicating higher fractional anisotropy and BDNF concentrations after tDCS procedures, which provide biological plausibility for the observed preliminary improvements [45]. Comparison with other meta-analyses demonstrates some consistency with findings in other neurological populations, though effect sizes vary. Similar motor improvements but with greater effect sizes were observed in the meta-analysis by [29] with tDCS in stroke patients, possibly reflecting differences in lesion patterns between stroke's focal pathology and MS's diffuse pathology.

In contrast, our preliminary cognitive outcomes appeared more pronounced than those reported by Hill et al. [22] in healthy samples. This suggests that tDCS may be particularly beneficial in conditions with existing neuroplasticity impairments, though this requires further validation. The safety profile observed in our review (98% completion rates, minimal side effects) aligns with comprehensive safety reports by Buchanan et al. [6], indicating that tDCS is a well-tolerated intervention across different populations.

The subgroup analysis revealed significant differential treatment responses across multiple Sclerosis (MS) subtypes and stimulation protocols, with relapsing–remitting MS (RRMS) patients demonstrating more consistent responses to tDCS interventions compared to progressive MS patients. Stimulation site-specific analysis revealed that primary motor cortex (M1) stimulation showed the most consistent effects on motor functions, left dorsolateral prefrontal cortex (DLPFC) stimulation appeared most effective for cognitive enhancement, and cerebellar stimulation showed potential for balance and postural control functions. However, the heterogeneity in responses across MS subtypes, limited subgroup-specific data in many studies, and variable effectiveness of different stimulation sites contributed to overall uncertainty in evidence quality, highlighting the need for larger, more standardized trials to establish definitive protocols.

## Study strengths and limitations

This study comprehensively analyses multiple outcomes across various MS subtypes and disease phases. Including both motor and cognitive assessments enables broader insight into tDCS effects among patients with MS. The research followed sound methodology according to PRISMA guidelines, with systematic quality evaluation using validated instruments (RoB 2.0 and ROBINS-I). However, several significant limitations must be acknowledged. Most critically, the meta-analyses were based on minimal numbers of studies per analysis (SDMT: 2 studies, BBS: 3 studies, Gait speed: 3 studies), which significantly limits the confidence that can be placed in the pooled results despite low heterogeneity in some analyses. The heterogeneity in stimulation parameters across studies, variability in outcome measures and assessment timepoints, and diversity in MS subtypes and disease duration between participants further complicate interpretation. Additionally, the small sample sizes in individual studies (5–120 participants) provided insufficient statistical power for robust subgroup analyses. The follow-up periods varied considerably across studies, with most providing only short-term assessment, making it impossible to evaluate the long-term sustainability of treatment effects. Furthermore, the restriction to English-language publications may introduce language bias, potentially excluding relevant trials published in other languages. Readers should also note that several qualitative findings in the Results section describe within-group improvements from baseline; efficacy conclusions should be based primarily on between-group comparisons (active vs. sham tDCS), which represent the controlled effect. Where individual study results are cited as within-group improvements, these should not be interpreted as evidence of treatment efficacy in isolation. Additionally, primary studies varied in their use of post-treatment scores versus change-from-baseline scores; where possible, between-group differences were used for meta-analysis, but this could not be confirmed for all included studies and may introduce a degree of inconsistency in the pooled estimates. Finally, the exclusion of studies with unavailable full text, regardless of the reason for inaccessibility, may have introduced selection bias if the results of excluded studies differed systematically from included studies.

## Study implications

The study findings have various implications for clinical practice and research. There is a need for enhanced integration of tDCS into comprehensive MS rehabilitation programs, particularly for cognitive symptoms. In addition, this study highlights the importance of protocol standardization, with evidence suggesting that multiple sessions combined with conventional therapy yield optimal results. Specifically, protocols involving 10–15 sessions delivered at 1.5–2.0 mA intensity for 20–30 min per session, targeting the left dorsolateral prefrontal cortex (DLPFC), have shown the most promising cognitive benefits. Combining tDCS with cognitive training or physical therapy appears to enhance treatment effects, highlighting the need for multimodal rehabilitation approaches. Moreover, it is essential to recognize the potential for home-based tDCS interventions, given remote supervision's demonstrated feasibility and safety. However, widespread adoption of home-based tDCS will require standardized safety monitoring and comprehensive patient training to ensure proper use. Findings of this review will shape future research, emphasizing the need for larger, multi-center randomized controlled trials to establish definitive protocols and the importance of long-term follow-up studies to understand durability of treatment effects.

## Conclusion

This systematic review and meta-analysis indicate that transcranial direct current stimulation (tDCS) may offer therapeutic benefits as a supplementary treatment for motor and cognitive symptoms in multiple sclerosis (MS) patients. The limitations of this evidence are small sample sizes in the individual analyses and study heterogeneity, which limit the strength of the evidence and the need to interpret the findings carefully. The synthesis of the existing literature shows initial positive changes in cognitive processing speed and functional mobility; however, these indicators are based on small data volumes and should be verified in large trials. The early results related to motor functional outcomes indicate promise to improve functional mobility, as expressed by the Timed Up and Go test. The pooled analysis of gait speed and balance was insignificant, and the limited number of contributing studies restricts confidence in motor benefits. In individual studies, motor improvements were reported consistently, so it may be necessary to use standardized procedures and even larger sample sizes to draw concrete conclusions regarding motor improvements. Notably, the side effects profile of tDCS in MS patients is favourable, and no serious adverse effects have been reported, nor have patients shown significant withdrawal rates across studies, indicating its feasibility as a safe non-invasive treatment option. In addition to motor and cognitive enhancing effects, these possible positive outcomes may converge into better independence, increased daily functioning, and quality of life, furthering the applicability of tDCS within the framework of comprehensive rehabilitation in MS.

The extensive study heterogeneity in stimulation parameters, outcome measures, participant characteristics, the small number of studies included in each meta-analysis, and the differences in the follow-up duration decrease confidence in these preliminary results. Most importantly, the lack of follow-up data does not allow for the evaluation of the durability of the treatment. The future should focus on standardised stimulation methods, implementation and use of core outcome sets, and sufficient follow-up data in large, multi-centred randomised controlled trials. Although the existing evidence is considered a source of further research, it should be treated as interim and exploratory, contrary to the final evidence of clinical effectiveness.

## Data Availability

Available upon the request.
